# Ultrasound-assisted hydrolysis of lard for free fatty acids catalyzed by combined two lipases in aqueous medium

**DOI:** 10.1080/21655979.2020.1729678

**Published:** 2020-02-24

**Authors:** Jinjin Huang, Qingyi Zhao, Wei Bu, Chunmei Zhang, Zhen Yang, Xin Zhang, Kaini Zhang

**Affiliations:** a Key Laboratory for Biotechnology on Medicinal Plants of Jiangsu Province, School of Life Sciences, Jiangsu Normal University, Xuzhou, P. R. China; b State Key Laboratory of Agrobiotechnology and MOA Key Laboratory of Soil Microbiology, College of Biological Sciences, China Agricultural University, Beijing, China

**Keywords:** Lard oil, free fatty acids, ultrasound, *Rhizomucor miehei* lipase, *Penicillium cyclopium* lipase

## Abstract

Lard is a by-product of animal processing. It is inexpensive compared with vegetable oils; however, its use is limited due to the high calorific value and high-saturated fatty acid content. While using lard as the source of free fatty acids (FFA) can significantly increase its utilization value. This study aimed to research the method on efficient hydrolysis of lard catalyzed by *combi*-lipases and assisted with ultrasound pretreatment. A 1,3-specific lipase from *Rhizomucor miehei* (termed pRML, 1540 U/mL) and a nonspecific mono- and diacylglycerol lipase from *Penicillium cyclopium* (termed MDL, 2000 U/mL) were used as biocatalysts. Results showed that the maximum hydrolysis rate of lard after 6 h at 45°C by using pRML and MDL alone was, respectively, 39.9% and 8.5%. When pRML combined with MDL (*combi*-lipases), hydrolysis rate can reach to 78.1%. While *combi*-lipases were assisted with 5 min ultrasound pretreatment before the reaction, the hydrolysis rate can further increase to 97%. The *combi*-lipases with different specificity and assisted with ultrasound pretreatment may be a useful technology for the enzyme production of FFA from complex lipid substrates, such as lard.

## Graphical Abstract

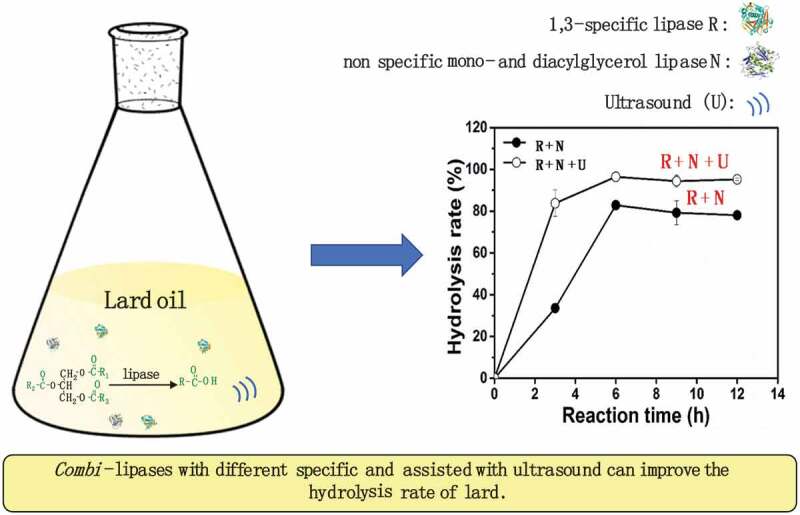

**Abbreviations:** FFA: free fatty acids; TAGs: triacylglycerols; SEs: steryl esters; WEs: wax esters; pRML: *Rhizomucor miehei* lipase; MDL: *Penicillium cyclopium* lipase; BMGY/BMMY: buffered complex glycerol/methanol medium; BSMG: base salt media with Na_2_GP as phosphate source; YNB: yeast nitrogen base W/O amino acids; GC: gas chromatography; TLC: thin-layer chromatography;

## 1. Introduction

Free fatty acids (FFA) can be converted into a broad range of biofuels and biochemical products through straightforward chemical reactions and are thus widely used in food, medicines, chemical and other industries [1–3]. FFA can be used directly or in the form of derivatives [4]. C8–C14 are mainly used in the production of surfactants and C16–C18 are used for the production of stearic acid esters, fatty acid salts, fatty acid methyl ester, cationic surfactants, and synthetic resins, which can be used as food additives and biodiesel [4–7].

FFA are commonly prepared through the chemical method, which uses natural oils or petroleum hydrocarbons (paraffin) as raw materials [8]. The chemical preparation of FFA causes environmental pollution and energy-intensive, because it used the method of alkali saponification and acidification, and reaction needed high temperature and pressure [8]. In recent years, research on the production of bio-oils by biological method has received increasing attention, especially the study of microbial oils [3–9]. Microbial oils can be produced by many microorganisms, including bacteria and fungi, such as yeast and microalgae [10]. Those microorganisms can store lipophilic compounds in the form of intracellular droplets, such as triacylglycerols (TAGs), steryl esters (SEs), and wax esters (WEs) [11]. Moreover, many studies have improved the ability of microorganisms to accumulate microbial oils through cultivation methods and genetic engineering techniques [12]. These studies have successfully improved total ester content with abundant TAGs, SEs, and WEs in the form of esterified FFA [13,14]. The esterified FFA also needs post-processing. Thus, attention has gradually shifted to the microbial production of FFA [3,15]. FFA is harmful to microorganisms because of their amphiphilic nature, but some microorganisms are now able to produce FFA through genetic engineering [9]. Those microorganisms include *Escherichia coli, Yarrowia lipolytica*, and *Saccharomyces cerevisiae* that can produce the following amounts of FFA: 8.6 (bioreactor), 10.4 (bioreactor), and 2.2 g/L (flask), respectively [9,16,17]. Nonetheless, the commercial production of microbial oils is limited to high-value products (docosahexaenoic acid, eicosapentaenoic acid, and gamma linoleic acid) because of the costly production and extraction process [9,10,12]. Considerable work, such as obtaining maximum yield, exploring simple and low-cost extraction methods, is needed to produce FFA through the use of microorganisms[9]. Among the known raw materials, natural oils have an indispensable advantage in the current industrialized fatty acid production.

Lard is a by-product of the processing of animal products and inexpensive compared with vegetable oils [18]. Lard is cheap, however, its use is limited due to its high calorific value and high-saturated fatty acid content [19]. The main ingredient of lard is TAG and can be used only to produce diacylglycerol, biodiesel, and 1,3-olein-2-palmitin [18,20,21]. From a different perspective, lard can be used as a source of green preparation FFA for more widespread application. Compared with chemical method, enzyme-catalyzed conversion reaction is environment friendly and becomes a ‘hot’ research topic [22]. The hydrolysis of vegetable oil (castor oil, olive oil, cottonseed oil, and palm kernel oil) with lipase as a catalyst is widely studied using free or immobilized lipase [23,24]. However, few studies research on enzymatic hydrolysis of animal fats, such as lard. Report found that the relative hydrolysis rate of lard to produce FFA was only 17.24% hydrolyzed by lipase from *Galactomyces geotrichum* [25]. The fatty acid composition of each oil is extremely complex, and the preference of the enzyme is different. Therefore, investigating the efficient enzymatic hydrolysis of lard is necessary.

In our previous study, 1,3-specific lipase from *Rhizomucor miehei* (termed pRML) and nonspecific mono- and diacylglycerol lipase from *Penicillium cyclopium* (termed MDL) were cloned and expressed in *Pichia pastoris*. The enzyme activity of pRML and MDL was 1200 U/mL (pRML, in flask by using buffered complex glycerol/methanol medium (BMGY/BMMY) medium), and 18,000 U/mL (MDL, in a 7.5 L fermenter by using base salt media with Na_2_GP as phosphate source (BSMG)), respectively [5,22]. In the present study, we changed the cultivation strategy to produce pRML and MDL, and combined the two lipases to catalyze lard for producing FFA assisted with ultrasound in an aqueous medium.

## 2. Materials and methods

### 2.1. Strains and culture conditions

pRML-produced strain constructed in a previous study [22], which was named mα-2pRML-X33 [22]. In the present study, the strain was cultured in BMGY medium as seed liquid, and when OD_600_ reached to 4–8, the seed was transferred to a 7.5 L autofermentor (Baoxing; Shanghai, China) containing 5 L BMMY medium with 1.34% (w/v) yeast nitrogen base W/O amino acids (YNB). Methanol was used to induce pRML expression. The fermentation method was similar to that in our previous study with the agitation rate of 700 rpm and aeration rate of 10 L/min at 28°C [26,27]. OD_600_ and pRML hydrolysis activity was previously reported and every sample was detected with three replicates [22]. The fermentation broth was centrifuged at 12,000 rpm for 5 min to remove the yeast cell, and the supernatant was directly used for catalytic reaction.

MDL-produced strain (GS115-MDL) was constructed by Guan et al. (2010) [26]. In this study, the strain was flask-cultured in BSMG medium. Seed liquid was cultured in a YPD medium at pH 7.0 with OD_600_ reached to 4–8, then transferred it to BSMG medium (pH 7.0) containing 0.4% (v/v) PTM1. The formula of BSMG and PTM1 was shown in Huang et al. (2013) [5]. The strain firstly entered to glycerol batch phase. After glycerin completely consumed, 0.5% (v/v) methanol was added to induce MDL expression, marking the methanol fed-batch phase. NH_4_OH (28%) was added every 24 h as nitrogen source and pH was adjusted to 7.0. The culture temperature was adjusted to 28°C. Cell density (estimated as OD_600_) and lipase activity were evaluated every 24 h, and three replicates were set up for every sample. The detection of MDL hydrolysis activity was reported in 2013 study [5]. Method of supernatant preparation was the same as pRML.

### 2.2. Lard extraction

Lard was purchased from RT-MART supermarket of Xuzhou. It was cut into small pieces of 1 cm, heated them in a pot, and removed the filter residue. The mixture oil was ﬁltered through four layers of gauze, cooled at room temperature, and stored at 4°C. The ﬁltered oil was used for the next experiment.

### 2.3. Qualitative and quantitative determination of fatty acids by gas chromatography

The sample was prepared as follows: sample was weighed in the hydrolysis tube. 4 mL chloroacetyl methanol solution (chloroacetyl: methanol = 1:10 (v/v)), 1 mL C11:0 (1.0 mg/mL) internal standard solution and 1 mL n-hexane were added to the tube. Then it was placed in a water bath at 80°C for 2 h with the cap covered. Finally, 5 mL potassium carbonate (5%) was added to stop the reaction and the mixture was centrifuged at 1000 rpm for 5 min, then loaded through 0.2 μm of filter membrane to the injection vial test.

The gas chromatography (GC) analysis conditions were as follows: the sample was analyzed using a GC system (model 6890, Agilent Technologies, Santa Clara, California, United States) equipped with a capillary column (DB-23; 60.0 m × 250 um × 0.25 um; Agilent Technology). The carrier gas was He with a flow rate of 2.0 mL/min and split ratio of 30:1. The column temperature was initially kept at 150°C for 1 min and increased to 190°C at 4°C per minute, maintained for 1 min. The temperature increased to 230°C at 20°C per minute and maintained for 5 min. The temperature of injector and detector were set at 260°C and 270°C, respectively. The Sample was detected three times.

### 2.4. Preparation mixed fatty acid from lard with or without ultrasonic treatment

The production conditions were as follows: 4 g lard, 600 U pRML and 75 U MDL per gram of lard, water content was 35.4wt%; for pRML alone, MDL was replaced by H_2_O. The reaction system was subjected to ultrasonic bath (SK250H, Kedao, Shanghai, China) for 5 min ultrasonic pretreatment (frequency US 53 kHz and ultrasound power of 250 W). Then the reaction system was placed in a water bath shaker at 30–50°C and shaken at 150 rpm for 12 h. Every reaction had three replicates. The sample was analyzed every 3 h.

### 2.5. FFA detection via NaOH titration and thin layer chromatography

The sample was obtained and centrifuged at 12,000 rpm for 10 min. Then, 200 µL of the upper layer was mixed with 3 mL hexane and 7 mL ethanol. Thereafter, 0.05 M NaOH was used to titrate the mixture with phenolphthalein as an indicator. The volume of NaOH consumed by 200 µL of oleic acid was used as the control for complete hydrolysis of lard. The hydrolysis rate of lard was calculated as V_sample_/V_oleic acid_. The hydrolysis rate was calculated for each replicate and the averaged data of the replicates were used for next analysis.

Thin layer chromatography (TLC) determination was performed in accordance with our previous study [28]. The upper layer (1 µL) was dissolved in 50 µL hexane, and 2 µL mixture was subjected to TLC silica gel plate for analysis. Thereafter, 20% (v/v) ethyl sulfate was used as the color reagent. The TLC plate was heated until the point appeared.

## 3. Results and discussion

### 3.1. pRML and MDL preparation

**Figure 1.**
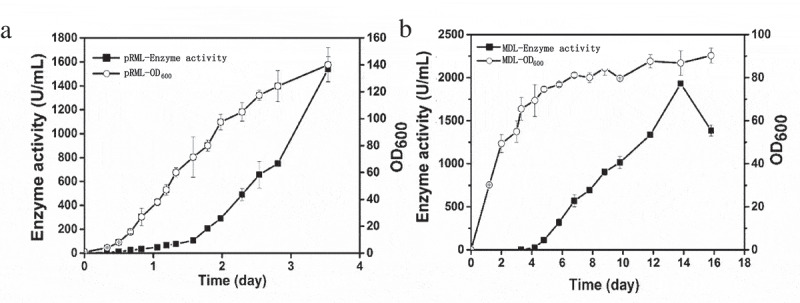
Cell density (OD_600_) and enzyme activity during pRML and MDL production. (a) pRML-produced strain cultured in 7.5 L autofermentor using BMGY/BMMY medium. (b) MDL-produced strain was achieved in flask fermentation using the BSMG medium. The highest pRML enzyme activity was 1540 U/mL and OD_600_ reached 140 at day 3.5 in 7.5 L autofermentor. The enzyme activity of MDL gradually increased and reached the maximum 2000 U/mL on the 14th day with the OD_600_ at 86.

In our previous study, a pRML-produced strain (mα-2pRML-X33) containing two copies of *prml* was constructed and cultured in BMGY/BMMY medium with enzyme activity (1200 U/mL) through shake flask fermentation [22]. In the present study, pRML production was investigated in a 7.5 L autofermentor, and BMGY/BMMY medium was also used. Cell growth (OD_600_) and enzyme activity were detected. The result was shown in Figure 1(a). In the BMGY/BMMY cultivation strategy, methanol was the only carbon source in BMMY medium during fermentation and an inducer of protein expression. Figure 1(a) shows that the lipase production of mα-2pRML-X33 can be gradually improved by increasing methanol induction time. The enzyme activity of pRML reached a maximum value at day 3.5. The highest pRML enzyme activity was 1540 U/mL, and OD_600_ reached 140 under 7.5 L autofermentor fermentation conditions (Figure 1(a)). The production of pRML by mα-2pRML-X33 in 7.5 L autofermentor (1540 U/mL) was higher than that previously reported in shake flask fermentation (1200 U/mL) and the fermentation time of mα-2pRML-X33 in 7.5 L autofermentor was shortened from 5 days to 3.5 days.

Different with the process in 7.5 L fermenter [5], the flask fermentation process of MDL-produced strain used BSMG medium can be divided into only two phases: glycerol batch and methanol fed-batch phase. The seed liquid of MDL-produced strain was cultured in YPD medium and was transferred to BSMG containing glycerin as the only carbon source. Glycerin allows *P. pastoris* to rapidly accumulate large amounts of biomass but inhibits protein expression [5]. Thus, no enzyme activity of MDL was detected in this phase, but OD_600_ reached 54 after 3.3 days (Figure 1(b)). The purpose of this phase is to accumulate biomass for methanol induction. When glycerol was completely consumed, methanol was added every 24 h to induce protein expression after 3.3 days. The enzyme produced at day 3.3 and gradually increased until the 14th day and reached the maximum of 2000 U/mL (Figure 1(b)). OD_600_ increased from 54 to 86 after 13.8 days during the methanol fed-batch phase (Figure 1(b)). The MDL production (2000 U/mL) achieved from the low-cost BSMG medium in a flask was higher than that reported in BMGY/BMMY medium (1500 U/mL) [26].

### 3.2. Oil extraction and FFA analysis of lard

**Figure 2.**
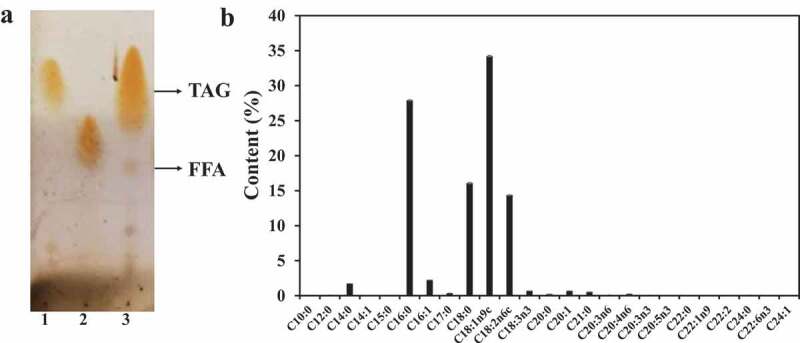
Lard composition. (a) TLC detection. Lane 1 TAG standard; Lane 2 FFA standard; Lane 3 lard; (b) GC detection. The main component of lard oil was TAG containing palmitic acid (C16:0, 27.9% m/m), stearic acid (C18:0, 16.1% m/m), oleic acid (C18:1, 34.2% m/m), and linolenic acid (C18:2, 14.4% m/m).

Nature oil is complex and has different fatty acid compositions [24]. The composition of lard was measured by TLC and the result found the main component of it was TAG and a small amount of FFA (Figure 2(a)). This finding was different from that for microalgae oil whose main component is FFA [28]. Fatty acid species in lard was qualitatively and quantitatively analyzed via GC, and the results are shown in Figure 2(b). The fatty acid content in 1 g of lard oil was 0.991 g (Table 1). The main components were palmitic acid (C16:0, 27.9% m/m), stearic acid (C18:0, 16.1% m/m), oleic acid (C18:1, 34.2% m/m), and linolenic acid (C18:2, 14.4% m/m) (Figure 2(b) and Table 1). These fatty acids accounted for 92.6% of total fatty acids (Table 1). The highest content component of lard is oleic acid (C18:1, 34.2% m/m), which is different from the highest content component of some vegetable oils, such as palm oil (C16:0, 52.7% m/m), olive oil (C18:1, 78.1% m/m), soybean (C18:2, 53.4% m/m), and waste frying oil (C18:1, 56.2% m/m) [29].

**Table 1.** Qualitative and quantitative determination of fatty acids in lard.

FFA	Content in lard (mg/g)	Content in total FFA (%)
C10:0	0.7 ± 0.014	0.1 ± 0.001
C12:0	1.0 ± 0.013	0.1 ± 0.001
C14:0	17.2 ± 0.149	1.7 ± 0.001
C14:1	0.2 ± 0.001	0.0 ± 0.001
C15:0	0.7 ± 0.006	0.1 ± 0.000
**C16:0**	**276.7 ± 1.383**	**27.9 ± 0.0781**
C16:1	22.2 ± 0.182	2.2 ± 0.001
C17:0	3.7 ± 0.0246	0.4 ± 0.000
**C18:0**	**159.5 ± 0.494**	**16.1 ± 0.076**
**C18:1n9c**	**339.3 ± 3.547**	**34.2 ± 0.091**
**C18:2n6c**	**142.4 ± 1.582**	**14.4 ± 0.048**
C18:3n3	6.8 ± 0.0814	0.7 ± 0.003
C20:0	2.3 ± 0.007	0.2 ± 0.001
C20:1	6.8 ± 0.080	0.7 ± 0.003
C21:0	5.3 ± 0.056	0.5 ± 0.001
C20:3n6	0.9 ± 0.013	0.1 ± 0.001
C20:4n6	2.4 ± 0.036	0.2 ± 0.002
C20:3n3	0.8 ± 0.021	0.1 ± 0.001
C20:5n3	0.1 ± 0.005	0.0 ± 0.001
C22:0	0.1 ± 0.006	0.0 ± 0.000
C22:1n9	0.3 ± 0.019	0.0 ± 0.002
C22:2	0.1 ± 0.003	0.0 ± 0.000
C24:0	0.8 ± 0.011	0.1 ± 0.000
C22:6n3	0.3 ± 0.003	0.0 ± 0.001
C24:1	0.4 ± 0.010	0.0 ± 0.001
**SUM**	**991.0 ± 7.730**	**100.0 ± 0.000**

Bold values are the main fatty acids of lard oil.

### 3.3. Combi-lipases (pRML and MDL) are better for FFA production in aqueous medium

Lipase with different specificities and selectivities is a unique enzyme that can catalyze a variety of reactions, such as hydrolysis, esterification, and alcoholysis [23]. Considerable attention has been given on vegetable oil hydrolysis using lipase, but research on the enzymatic hydrolysis of lard is relatively rare [23]. Combined with the characteristics of lard substrate (main component is TAG) with the specificities and selectivities of lipase, 1,3-specific lipase pRML and nonspecific mono- and diacylglycerol lipase MDL were selected to catalyze the hydrolysis of lard for FFA production. The fermentation supernatant was directly used for catalytic reactions.

**Figure 3.**
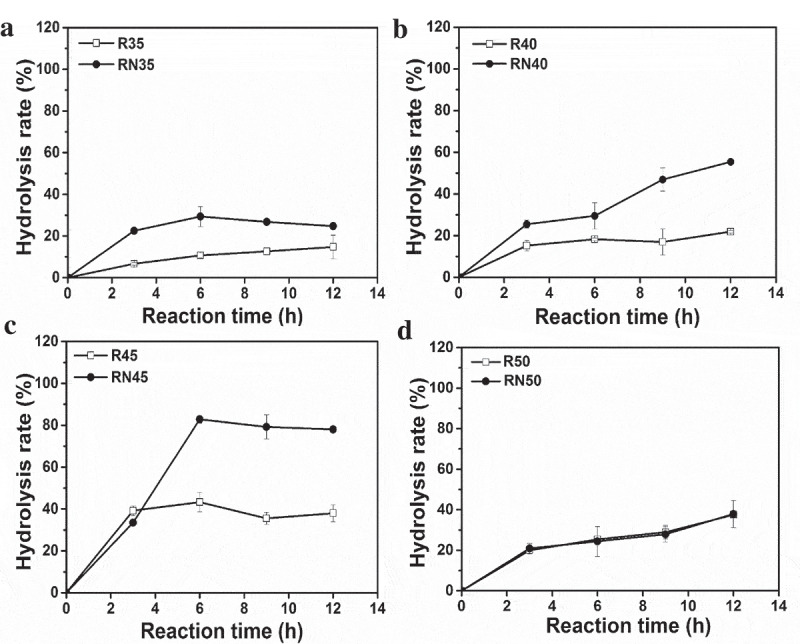
Effect of temperature on the hydrolysis of lard by lipase. (a–d) pRML only (R) and combination of pRML and MDL (RN) at 35°C, 40°C, 45°C, and 50°C. The combined lipases performed better than the single lipase at other reaction temperatures in addition to 50°C. Whether single or *combi*-lipases, the hydrolysis rate reached the maximum (R: 39.9% and RN: 78.1%) at 45°C after 6 h reaction.

The single enzyme (R) and combined enzyme (RN) catalytic reaction were performed. The reaction temperature was optimized from 35°C to 50°C. The result is shown in Figure 3. The efficiency of lard hydrolysis through a single enzyme and combined enzymes increased as reaction temperature increased from 35°C to 45°C (Figure 3(a–c)). Except at 50°C, combined enzymes were better than a single enzyme at other reaction temperatures (Figure 3(d)). Regardless of the number of enzymes used, hydrolysis rate reached its maximum at 45°C after 6 h of reaction. The maximum hydrolysis rate of combined enzymes and single enzyme were 78.1% and 39.9%, respectively (Figure 3(c)). The hydrolysis effect in both cases was the same at 50°C. Result also found hydrolysis rate of MDL alone was only 8.5% at 45°C after 6 h (Figure S1).

The *combi*-lipases Lipozyme RM-IM (RML, 1,3-regio-specific lipase) and Novozym 435 (CALB, no-specific lipase) were studied as biocatalysts to hydrolyze soybean oil, and 80% hydrolysis rate was achieved in 24 h [30]. This result was higher than that of any individual lipase [30]. However, the hydrolysis rate of soybean oil catalyzed by CALB alone, *combi*-lipases of Lipozyme TL-IM (TLL, 1,3-regio-specific lipase) and CALB, and *combi*-lipases RML and TLL did not improve [30]. This result may be attributed to substrate specificity. Not all the *combi*-lipases can increase the hydrolysis rate of complex oil. However, this study design of a *combi*-lipases which combined 1,3-regio-specific lipase (pRML) with on-specific mono- and diacylglycerol lipase (MDL) can improve the hydrolysis rate of lard from 39.9% (pRML alone) to 78.1% (*combi*-lipases) (Figure 3(c)). This enzyme combination is suitable for the hydrolysis of lard.

### 3.4. Ultrasound pretreatment accelerates reaction rate and improves hydrolysis efficiency

The *combi*-lipases of pRML and MDL can effectively improve the hydrolysis rate of lard to 78.1%, but the remaining 21.9% of lard should be addressed. Studies have found that ultrasound is a promising method for enzymatic synthesis [31,32]. Ultrasound pretreatment promoted diacylglycerol production from lard through lipase-catalyzed glycerolysis and does not change the structure of lard [19]. Thus, ultrasound was used in this study to improve the hydrolysis rate of lard catalyzed by *combi*-lipases.

**Figure 4.**
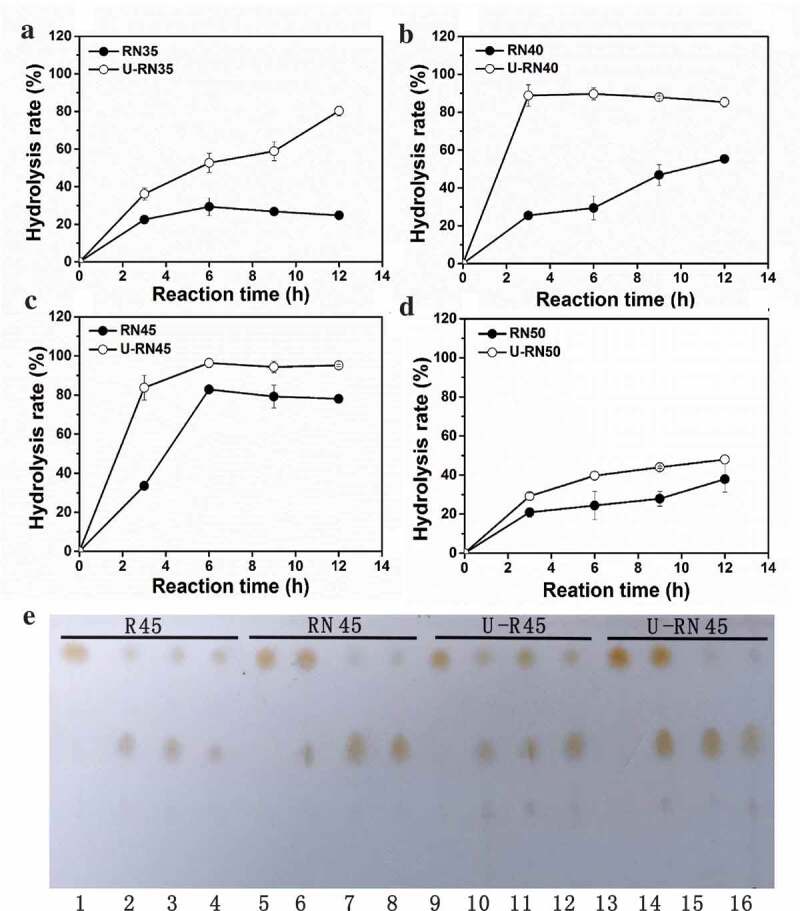
Effect of ultrasonic pretreatment on the hydrolysis of lard by *combi*-lipases at different reaction temperatures. (a–d) Reactions at 35°C, 40°C, 45°C, and 50°C; E: Reaction mixture samples detected by TLC. U-RN represented the combined enzyme under ultrasonic pretreatment. 5 min of ultrasonic pretreatment can improve the hydrolysis rate of lard by ~20% to 97% at 45°C after 6 h of reaction.

The catalytic reaction systems of a single enzyme and combined enzymes were the same as above, but we extended the ultrasonic pretreatment by 5 min to investigate its effect on the enzymatic hydrolysis of lard at reaction temperatures of 35–50°C. The result is shown in Figure 4 and Figure S2. After 5 min of ultrasonic pretreatment, the hydrolysis rate of lard was no obvious improvement when catalyzed by pRML alone (Figure S2), while all increased when catalyzed by *combi*-lipases at temperature 35–50°C (Figure 4). Assisted with ultrasonic treatment, the hydrolysis rate at 40°C and 45°C reached to 88% and 89% after 3 h of reaction catalyzed by *combi*-lipases, respectively (Figure 4(b,c)). But the maximum hydrolysis rate reached 97% at 45°C after 6 h (Figure 4(c)). The maximum hydrolysis rate improved by 18.9% after ultrasonic pretreatment at 45°C. Although the hydrolysis rate improved at 50°C after ultrasonic pretreatment, it was only 40% after 6 h (Figure 4(d)). The reaction mixture taken at different times with a single enzyme and combined enzyme with or without ultrasonic pretreatment at 45°C was detected by TLC. The result is shown in Figure 4(e). FFA production increased as the reaction proceeded, and lard was almost completely hydrolyzed when catalyzed by *combi*-lipases with ultrasonic pretreatment at 45°C and 6 h of reaction (Figure 4(e)).

Low-energy ultrasound in enzymatic reaction has attracted considerable attention in recent years, and been demonstrated to modify temperature and pressure in microenvironments because of its cavitational effect [33]. The cavitation phenomena caused by ultrasound can enhance substrate dissolution, improve mass transfer, change protein conformational and perturb weak interactions, thereby increasing the rate of product formation [34,35]. We found that 5 min ultrasonic pretreatment can improve the hydrolysis rate of lard by ~18.9% at 45°C. The hydrolysis rate of *combi*-lipases increased at 35°C −50°C maybe owing to the cavitation phenomena caused by ultrasound. This finding also proved that ultrasound is beneficial to *combi*–lipases hydrolysis reaction. Thus, ultrasound-assisted for lard hydrolysis catalyzed by the combined two lipases with different specificities is a useful green technology for FFA production.

## 4. Conclusions

pRML and MDL were combined to hydrolyze lard for the preparation of FFA. The aim was to increase the utilization value of lard. TLC and GC analysis results showed that the main component of lard was TAG and 1 g of pork oil contains 0.991 g of FFA with palmitic acid (C16:0, 27.9% m/m), stearic acid (C18:0, 16.1% m/m), oleic acid (C18:1, 34.2% m/m), and linolenic acid (C18:2, 14.4% m/m). The *combi*-lipases catalytic reaction was assisted by ultrasound pretreatment with 600 U of pRML and 75 U MDL per gram of lard, and water content of 35.4 wt%. The hydrolysis rate reached its maximum (97%) at 45°C after 6 h. The ultrasound-assisted hydrolysis of lard for FFA production catalyzed by combining two lipases with different specificities in aqueous medium is a useful technology for complex substrate hydrolysis.

## Supplementary Material

Supplemental Material

## Highlights

1. High lipase activity of pRML (1540 U/mL) was obtained in 7.5 L autofermentor.
2. Combined pRML and MDL can improve the hydrolysis rate of lard up to 78.1%.
3. Ultrasound-assisted *combi*-lipases improved the hydrolysis rate of lard to 97%.
