# Supplementary material for: Ultrasound-assisted hydrolysis of lard for free fatty acids catalyzed by combined two lipases in aqueous medium
Source: Bioengineered. 2020 Feb 24;11(1):241–50. doi: 10.1080/21655979.2020.1729678 (PMC7039637; doi:10.1080/21655979.2020.1729678)
Supplement: Supplemental Material [file kbie-11-01-1729678-s001.docx]

**Figure S1**

**Figure S1** Hydrolysis of lard by MDL at 45 °C.

MDL cannot catalyze hydrolysis of lard to produce FFA.

**Figure S2**

**Figure S2** Effect of ultrasonic pretreatment on the hydrolysis of lard by pRML alone at different reaction temperatures.

The hydrolysis rate of lard was no obvious improvement when catalyzed by pRML alone after 5 min of ultrasonic pretreatment.
